# A mixed methods study of using wasta in healthcare services in Palestine: predictors, consequences and proposed solutions

**DOI:** 10.1186/s12913-023-10114-5

**Published:** 2023-10-19

**Authors:** Adel Takruri, Inad Nawajah, Carol El Jabari

**Affiliations:** 1 Grants Office, Hebron University, Hebron, Palestine; 2 Faculty of Science, Hebron University, Hebron, Palestine; 3 College of Nursing, Hebron University, Hebron, Palestine

**Keywords:** Wasta, Healthcare, Equity, Quality, Palestine

## Abstract

**Background:**

Equity in access to quality healthcare is a fundamental human right. Yet studies demonstrate that some people receive preferential treatment while others are discriminated against. Wasta is a prevalent strategy whereby personal connections are used for influence and may result in gaining unfair advantages over others. This study aims to investigate wasta use in healthcare, the factors associated with its use, and the impact of wasta use on the equity and quality of healthcare services.

**Methods:**

A mixed-methods study utilizing a quantitative survey and qualitative interviews was conducted in Palestine (West Bank and Gaza) between October 2021 and February 2022. Quantitative analysis was performed using Stata version 14. Bivariate and multivariate logistic regressions assessed the relationship between wasta use and individual-level variables such as gender, residence, age, employment status, and financial situation. Content analyses of qualitative transcripts were performed using Dedoose version 9. Textual quotes were grouped into major and minor themes.

**Results:**

Multivariate regressions revealed that wasta use is more prevalent among refugee camp dwellers and more frequent in Gaza compared to the West Bank. Wasta was also employed to a greater degree among government employees. Qualitative interviews complemented the quantitative results and added further insights into the consequences of Wasta use in healthcare settings such as negatively impacting quality and equity in healthcare services.

**Conclusion:**

Wasta use in healthcare can have an adverse effect on equity and quality. Ensuring efficient processes, reduced financial burdens, stringent accountability measures, transparency, and training programs can contribute to diminishing the need for using wasta in healthcare. By addressing both systemic and cultural factors that perpetuate wasta, societies can move closer to healthcare systems characterized by fairness, accessibility, and ethical integrity.

**Supplementary Information:**

The online version contains supplementary material available at 10.1186/s12913-023-10114-5.

## Introduction

Impartiality in healthcare access and provision contributes to quality and ethical healthcare delivery [1]. A major hurdle to achieving equity in healthcare access and delivery in Palestine is the use wasta or favoritism to gain access to services and privileges. Alsarhan & Valax (2020) define wasta as “*an unwritten social contract based on the cooperation and obligation between members of various social groups such as families and tribes”* [2, p.1]. The Arabic word “wasta” literally means mediation or intercession. Wasta, therefore includes an actor who intercedes or mediates on behalf of someone else; i.e. an intermediary who intercedes or mediates on behalf of someone else [3]. However, our interviews revealed that using one’s own clout to gain advantages is also considered wasta. Unlike bribery, which involves exchanging favors for money, wasta involves providing favors through social networks [4].

Similar social processes are also known in other cultures. In the West, the terms *social capital* and *networking* are used to obtain social advantages although not thought of negatively. Likewise, *guanxi* in China [5], *blat* in Russia, and *pulling strings* in the USA – are similar concepts with nuanced differences [6]. Unlike social capital and networking in the West which are seen as a desirable advantage, wasta in the Arab world is seen as essential for achieving one’s goals [7].

The mediators of wasta can be direct relationships such as family members or tribal associates. Mediators can also be people not in direct familial or tribal relationships but with whom loyalties have been cultivated such as friends, colleagues, religious groups, social clubs, and political parties [8]. Wasta can influence everything from simple actions such as skipping lines to more significant matters such as securing employment, receiving job promotions, or even obtaining preferential treatment within hospitals. In Palestine, wasta is often colloquially and sarcastically referred to as *‘vitamin W.‘* A deficiency in this “*vitamin*” is thought to impede one’s chances of promotion and advancement in various spheres of Palestinian society. The pervasiveness of wasta is rooted in strong family ties, tribal attachments, and group loyalties which necessitate providing assistance and preferential treatment to within-group members [4].

### Predictors and consequences of wasta

In countries where official processes to reach a goal are corrupt, bureaucratic, or inefficient, individuals resort to wasta to overcome oppressive processes [9]. Certain cultural contexts allow for wasta to propagate and fester. Patriarchy, tribalism, religious and political strife, inequities, discrimination, and lack of trust in public institutions are contextual vices within which wasta proliferates [5, 10]. On the other hand, accountability, transparency, and the rule of law reduce the need for wasta [10].

Wasta results in inequalities by depriving women and those with lower social networks from accessing opportunities [6, 11]. Those who have wasta may get access to services that are denied to those who do not have wasta, or they may have faster access [8]. Additionally, wasta encourages people to invest more in social networks rather than other more useful forms of social capital [7]. As a result, seniority, position, and status become more important than performance and abilities [5, 12]. The worst part of wasta is that it favors the rich and powerful at the expense of the vulnerable and poor, a sort of affirmative action for the advantaged [4].

While the role of wasta in healthcare is understudied, it is likely that the presence of wasta in a community could impact the behaviors of patients, providers, and healthcare organizations. Regarding patient behavior, wasta may undermine trust in the healthcare system. Trust in turn has been associated with seeking healthcare, compliance with treatment, and better communication with healthcare providers [13]. Consequently, wasta could decrease the utilization of healthcare services via a lack of trust in the system. Additionally, wasta may lower healthcare providers’ motivation, satisfaction, and morale. When employee recruitment and promotion are based on wasta, those without wasta will lose their motivation to perform and improve their work. This will affect employees with wasta as well since peers will connect career advancement with wasta and not performance [2]. Loyalty and commitment to the organization will eventually decrease.

In addition to lowering providers’ motivations to excel, wasta diminishes organizational performance by promoting less qualified personnel over more qualified ones. In a qualitative study by [2, p.9], one participant said: *“Imagine recruiting unqualified persons to lead an institution which has employees more qualified than their leader. The unqualified leader will lower the quality of performance and productivity and the morale of people under his management.*” Across settings, including healthcare, wasta perpetuates corruption, inequalities and unfairness, resulting in the allocation of resources based on relationships rather than by rules and principles [7, 14].

Despite the negative consequences of wasta, some people wrongly perceive wasta as a form of social capital whereby its use strengthens relationships and friendships and fosters bonds, loyalties, and trust [15]. It is true that within a networking perspective, wasta can strengthen belonging to a social group, promote solidarity, and enhance loyalty [8]. However, this positive interpretation often overlooks the fact that wasta can also perpetuate inequality, create unfair advantages, and undermine the merit-based systems that should ideally govern various aspects of society.

### Study setting

**Fig. 1:**
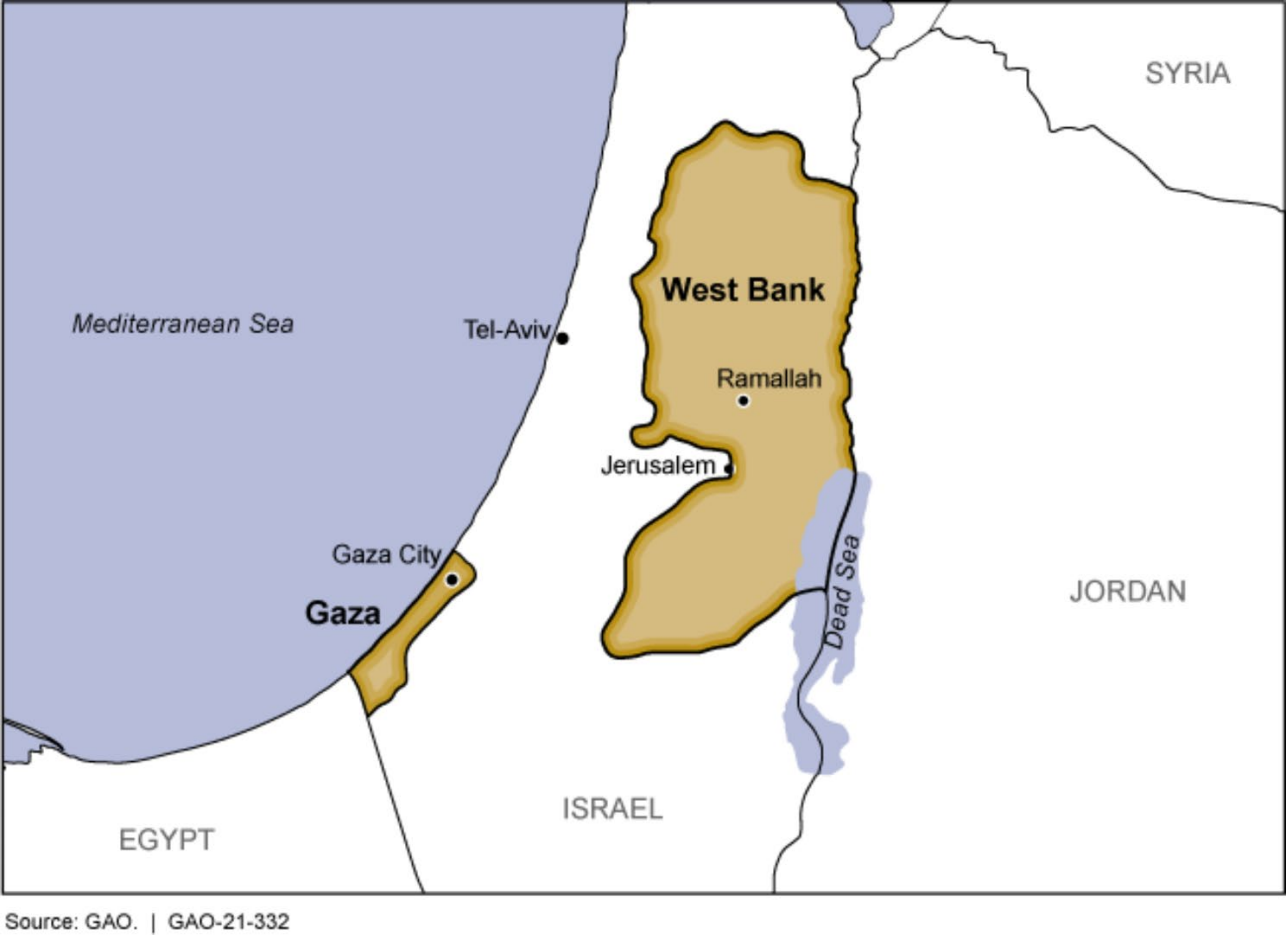
Map of the occupied Palestinian territories showing its division into 2 regions: Gaza and West Bank Source: https://www.gao.gov/products/gao-21-332

The occupied Palestinian territory (oPt) is a low-to middle-income country (LMIC) with a GDP per capita of USD 3,664 in 2021 [16]. It is divided into two separate regions, the West Bank which lies inland and the Gaza Strip which lies on the Mediterranean Sea (Fig. 1). According to the latest Palestinian Central Bureau of Statistics [17], the total population in the West Bank was approximately 5 million while Gaza had a population of 3 million.

The total unemployment rate in Palestine for individuals aged 15 years and above in 2022 was 24.4%. This total unemployment rate, however, conceals massive disparities by gender and region. Unemployment rates are notably higher in Gaza (45.3%) than in the West Bank (13.1%). Unemployment rates are also considerably higher among females (40.4%) than among males (20.3%) [17]. The unemployment rates among health professionals are around the national average (24.7%) [18].

Access to healthcare can be hampered by several factors. These include: (1) geographical obstacles or long distances to the nearest healthcare center, (2) Israeli restrictions on movement [19], (3) high costs of healthcare, (4) long waiting times, and (5) fear of being misunderstood or mistreated by healthcare professionals [20]. Access is also prevented if the treatment is not available. Some advanced therapies, procedures, and surgeries are not available in the West Bank and Gaza. To overcome this limitation, the Ministry of Health may refer patients to nearby countries, such as Jordan, Egypt or Israel, where patients reported higher service quality [21].

In addition to the medical services provided by the government, nongovernmental organizations (NGOs) are also active in the Palestinian Territories. These include national NGOs such as the Palestinian Medical Relief Society (PMRS), the Gaza Community Mental Health Program, and the Health Works Committee (HWC). International NGOs that provide healthcare services include the International Medical Corps, Médecins Sans Frontières, the Red Crescent Society, and the United Nations Relief and Works Agency (UNRWA), which provides medical services for Palestinian refugees.

The majority of the studies cited above have investigated the use of wasta in general or in employment. Additionally, most studies rely on qualitative research. The aim of this study, therefore, is to explore individual factors associated with the use of wasta to obtain healthcare services using quantitative data analysis, and to supplement the quantitative analysis with qualitative interview data to further explore the causes and consequences of wasta use in healthcare. To the best of our knowledge, this is the first study to investigate wasta in healthcare settings utilizing a mixed-methods approach. Understanding the prevalence of wasta use in healthcare, along with its predictors and consequences will help policy makers, health administrators, and stakeholders recognize the issue and enact policies and strategies to address it. Ultimately this leads to more equitable and fair healthcare services.

## Methods

This study included a cross-sectional quantitative survey and qualitative interviews. Data collection took place in the West Bank and Gaza Strip between October 2021 and February 2022. The qualitative interviews aimed to complement and enrich the quantitative findings. Ten health professionals were interviewed to compare and triangulate their perspectives with those of the patients. The health professionals included two physicians, one physiotherapist, two head nurses, and five staff nurses. All health professionals were from government hospitals.

### The quantitative survey

#### Survey population and sample

Previous surveys have indicated that a sample of 792 households in the West Bank and 528 in Gaza adequately represent the Palestinian population [22]. Therefore, our goal was to obtain at least that number of surveys. A convenience sampling strategy was selected to maximize cost-effectiveness given the geography, time, COVID-19 pandemic and political constraints. Participants who were at least 18 years old, had utilized a healthcare service within the 12 months prior to the survey, were capable of participating, and provided verbal consent were included in the study.

#### Survey data collection

Data collectors were recruited, trained, and deployed throughout the West Bank and the Gaza Strip. They approached participants from public places, obtained verbal consent, and used tablets (notebooks) with an online survey link created using Survey Monkey (SurveyMonkey Inc., San Mateo, CA, USA; www.surveymonkey.com) to administer the questionnaire. The survey included 60 questions in Arabic and required approximately 20 min to complete. The research team ensured data collection quality by conducting regular field visits and applying logical checks to the entered data.

The survey questions included the outcome variable: Over the past year, did wasta help you get a health service? The response options for this question were “*yes*” and “*no”.* The survey also included individual level demographic questions: age, sex, residence (*City, Village, Town, Camp*), region (*West Bank, Gaza*), education (*none, elementary/secondary, Diploma, Bachelor, Master or doctorate*), employment (*student, government employee, nongovernmental employee, private business, unemployed/housewife/retired*), and financial situation (*very difficult/difficult, moderate, good/very good*).

#### Survey data analysis

The relationship between the outcome variable and the predictors (individual-level demographic questions) was assessed using three models:

Model I: Bivariate regression representing the relationship between use of wasta and each independent variable separately.

Model II: Multivariate logistic regression to predict the odds of using wasta by independent variables in the same model to control for confounding.

$$\displaylines{Ln\left( {\frac{y}{{1 - y}}} \right) = {\beta _0} + {\beta _1}sex + {\beta _2}age + {\beta _3}education + \cr {\beta _4}residence + {\beta _5}region + {\beta _6}employment{\text{ }}status + \varepsilon \cr}$$

Model III: Multivariate logistic regression with adding region* employment status interaction to assess whether the relationship between use of wasta and employment status varies by region.

$$\displaylines{Ln\left( {\frac{y}{{1 - y}}} \right) = {\beta _0} + {\beta _1}sex + {\beta _2}age + {\beta _3}education + \cr {\beta _4}residence + {\beta _5}region + {\beta _6}employment{\text{ }}status{\text{ }} + \cr {\beta _7}region*employment{\text{ }}status{\text{ }} + \varepsilon \cr}$$

where.

Y is the outcome variable representing use of wasta to access a healthcare service in the past year.

${\beta }_{0}$ is the intercept, the slopes ${\beta }_{1},\dots , {\beta }_{6}$are the coefficients associated with each independent variable and $?$ is the error term.

The level of significance was set at p value ≤ 0.05. Data analysis was conducted with Stata software version 14.

### The qualitative interviews

#### Interview participants

For the qualitative patient interviews, the study included 18 patients. These interviews incorporated open-ended questions and lasted between 45 and 60 min. Participants were purposively sampled, with 5 interviews conducted in Gaza and 13 in the West Bank. The selection criteria included patients who had experienced significant health issues requiring healthcare access. Patient ages ranged from 25 to 81 years, encompassing individuals with various conditions such as infectious diseases (e.g., COVID-19), cancer, and chronic diseases (such as diabetes and hypertension).

Interviewees were chosen based on their mental capacity to provide rich and detailed information about their healthcare experiences. The selection process also aimed to capture a diverse range of perspectives, ensuring the inclusion of individuals with varying experiences. The interviews were conducted individually, either in-person or over the phone. During lockdown periods caused by COVID-19, some interviews were also carried out via Zoom. The study also involved interviews with 10 healthcare professionals.

#### Interview data collection

The patients were asked to describe their experiences with the healthcare centers they visited in terms of the healthcare providers, challenges and difficulties they faced, and how they cope with the difficulties they face as a result of their health condition. The research team and trained data collectors conducted the interviews. The patient semi-structured interview guide is provided as a supplement to this article. Qualitative data collection and analyses were conducted iteratively [23, 24]. As the process unfolded, specific themes emerged, necessitating deeper exploration. To delve further into these themes, additional questions, prompts, and probes were incorporated into the interview guide.

#### Interview data analysis

Transcripts were entered into Dedoose version ***9.0.17*** (www.dedoose.com), a specialized computer program designed for qualitative data analysis. The transcripts were read line-by-line, and each line was given a code in an open coding manner. The codes were then organized into major themes and subthemes. Themes were allowed to emerge organically [25–27].

The research team members conducted analyses independently and then met and discussed the themes to ensure credibility and trustworthiness. Data were collected until theme saturation was achieved. The major and minor themes were as follows: (1) factors employed in using wasta: social status, social connections, family ties, political affiliations, and wealth; (2) predictors of wasta: cultural factors, lack of resources, and institutional policies; and (3) consequences of wasta: lowered employees’ motivation to perform, lowered quality of health services, and inequities of healthcare access.

## Results

### Quantitative survey

A convenience sample of 2165 individuals was recruited to participate in the survey, of whom 1299 agreed to participate, representing a 60% response rate. The characteristics of the survey sample are detailed in Table 1. Females, n = 703, accounted for 58% of the respondents. More participants were from the West Bank and most were city and town dwellers. The majority of participants, n = 744 (61.4%) were in the 18–39 age group, while those aged 40 and above accounted for n = 468 (36%) of the sample.

Education levels varied, with n = 553 (45.6%) holding a bachelor’s degree and n = 72 (5.54%) reporting having no formal education. At least n = 441 (36.4%) of participants reported being unemployed or retired. Regarding the financial status, most reported a moderate financial status, some reported a difficult or very difficult status and only n = 198 (16.3%) reported a secure financial status. A total of 412 (34%) of the respondents reported using wasta to obtain a health service in the last year (39% in Gaza vs. 31.6% in the West Bank).

**Table 1:** Sample characteristics, N = 1212

Gender	N	%
*Male*	509	*42.0*
*Female*	703	*58.0*
**Region**		
*West Bank*	835	*68.9*
*Gaza*	377	*31.1*
**Residence**		
*City*	629	*51.9*
*Village*	282	*23.3*
*Town*	112	*9.2*
*Camp*	189	*15.6*
**Age**		
*18–39*	744	*61.4*
*40–59*	334	*27.6*
*60 or above*	134	*11.1*
**Educational level**		
*None*	72	*5.9*
*Elementary/secondary*	316	*26.1*
*Diploma*	150	*12.4*
*Bachelor*	553	*45.6*
*Master or doctorate*	121	*10.0*
**Work**		
*Student*	126	*10.4*
*Government employee*	367	*30.3*
*Nongovernmental employee*	104	*8.6*
*Private business*	174	*14.4*
*Unemployed/housewife/retired*	441	*36.4*
**Financial situation, ability to meet the family monthly demands**		
*Very difficult/difficult*	329	*27.1*
*Moderate*	685	*56.5*
*Good/very good*	198	*16.3*
**Over the past year, did wasta help you obtain a health service?**		
*Yes*	412	*33.9*
*No*	800	*66.1*

Table 2 shows the relationship between wasta use in healthcare and background variables in the three models. The bivariate statistics in model 1 show that use of wasta is not associated with sex or age. The use of wasta increases with education but only becomes marginally significant among respondents with master’s education or above who are more likely to use wasta compared to those with no education (OR = 1.83, p = 0.057). Similarly, wasta use increases as the financial situation improves OR = 1.48, p = 0.037. The place of residence is strongly associated with the use of wasta. Those who live in camps are 1.77 times more likely to use wasta than those who live in cities, p = 0.001, and respondents from Gaza are 1.40 times more likely to use wasta than respondents from the West Bank, p = 0.009. Government employees are at higher risk of using wasta than others.

The multivariate logistic regression in model 2 shows that after adjusting for confounding, only place of residence and employment status remain statistically significant. The financial situation and education became nonsignificant, indicating that the relationship between those variables and use of wasta is a function of employment. Employed people are more likely to use wasta than students or unemployed people. Government employees are 1.71 times more likely to use wasta than unemployed persons, p < 0.001. The places of residence remained statistically significant in the multivariate analysis.

**Fig. 2:**
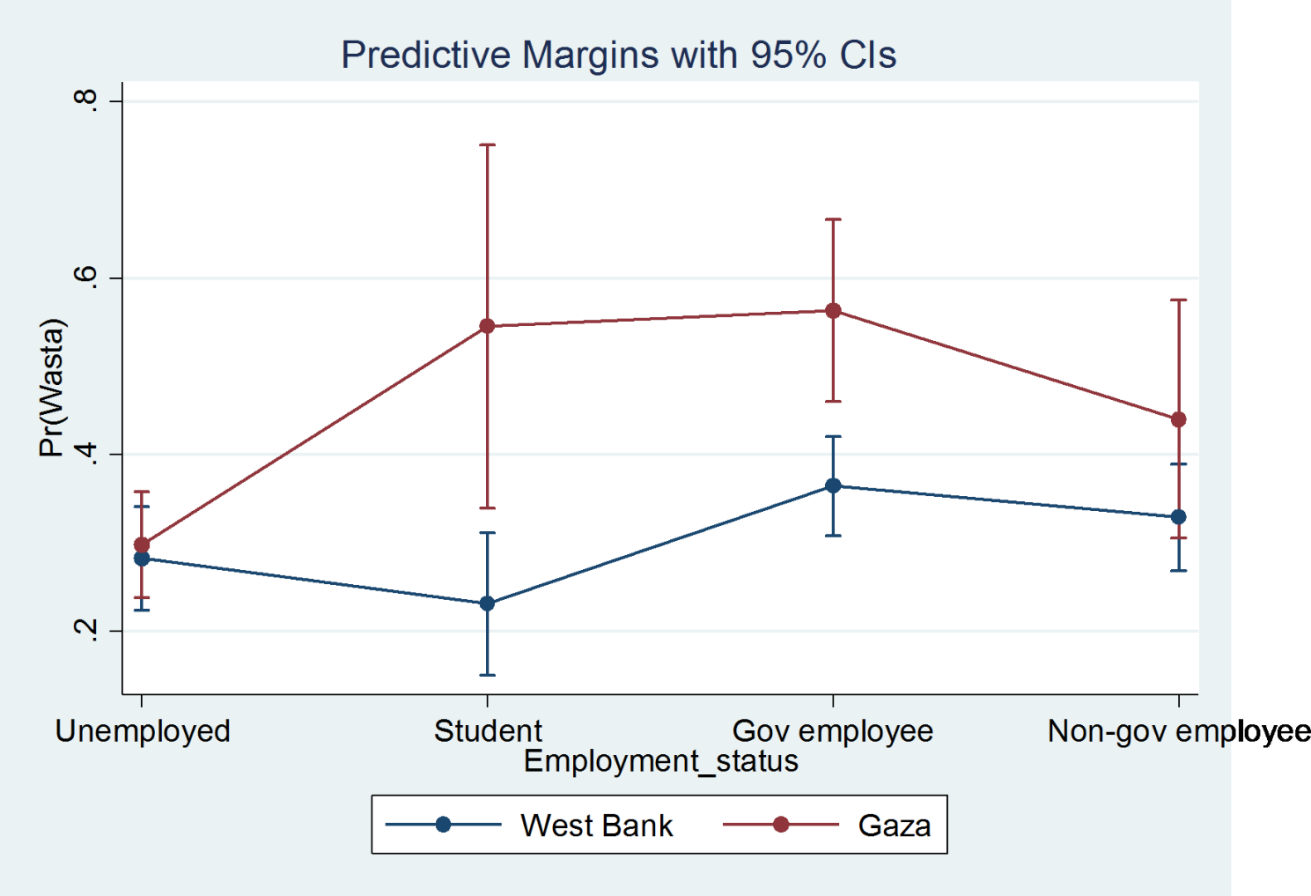
The relationship between use of wasta and employment status by region

Model 3 in the table adds a 4 by 2 interaction term between the 4-level employment status (unemployed, student, government employee, and nongovernment employee) and the 2-level region variable (Gaza, West Bank). The interaction coefficients are statistically significant and depicted in Fig. 2. The figure shows that respondents from Gaza are more likely overall to use wasta than respondents from the West Bank but the difference is mostly among students and government employees who are significantly more likely to use wasta in Gaza than students and government employees in the West Bank. The increased likelihood of using wasta among students compared to the unemployed is higher in Gaza (OR = 2.69) compared to the same relationship in the West Bank (OR = 0.66). The difference between those two ratios is statistically significant (2.69/0.66 = 4.06, p = 0.01).

**Table 2:** Odds ratios from logistic regression of wasta use by demographic characteristics, N = 1212

	Model 1 Bivariate		Model 2 Multivariate		Model 3 Multivariate with interaction	
	OR	P value	OR	P value	OR	P value
**Sex**						
*Male*	*Ref*		*Ref*		*Ref*	
*Female*	0.99	0.905	1.14	0.355	1.13	0.385
**Age**						
*18–39*	*Ref*		*Ref*		*Ref*	
*40–59*	0.98	0.903	0.93	0.648	0.90	0.498
*>=60*	0.74	0.147	0.92	0.709	0.88	0.573
**Education**						
*None*	*Ref*		*Ref*		*Ref*	
*Primary/secondary*	0.98	0.950	0.95	0.860	0.95	0.855
*Diploma*	1.18	0.599	1.00	0.989	1.00	0.992
*Bachelor*	1.37	0.256	1.18	0.597	1.21	0.526
*Master or above*	1.83	**0.057**	1.32	0.434	1.33	0.426
**Financial situation**						
*Difficult*	*Ref*		*Ref*		*Ref*	
*Moderate*	1.05	0.732	0.95	0.732	0.89	0.446
*Good*	1.48	**0.037**	1.35	0.145	1.29	0.220
**Residence**						
*City*	*Ref*		*Ref*		*Ref*	
*Village*	1.10	0.541	1.22	0.235	1.23	0.213
*Town*	0.90	0.648	0.95	0.841	0.94	0.806
*Camp*	1.77	**0.001**	1.68	**0.006**	1.67	**0.008**
**Region**						
*West Bank*	*Ref*		*Ref*		*Ref*	
*Gaza*	1.40	**0.009**	1.52	**0.014**	0.93	0.926
**Employment status**						
*Unemployed*	*Ref*		*Ref*		*Ref*	
*Student*	0.98	0.921	1.05	0.854	0.66	0.165
*Gov employee*	1.71	**0.000**	1.80	**0.002**	1.29	0.256
*Nongov employee*	1.31	0.099	1.53	**0.028**	1.22	0.382
Region*Employment status						
*Gaza # Student*					4.06	0.010
*Gaza # Gov employee*					2.28	0.014
*Gaza # Nongov employee*					1.55	0.262

### Results - qualitative interviews

Our interviews enriched our understanding of the individual-level factors associated with wasta use that were identified in the quantitative analysis, unveiled more factors related to wasta, and provided deeper insights into the nuanced dynamics of its utilization. Furthermore, the interviews also showed that in addition to using wasta for gaining privileges as patients, wasta is also used within healthcare institutions by some employees to gain unfair advantages over others.

Our interviews also highlighted social and organizational-level predictors and consequences of wasta. The potential predictors include scarcity of resources (material, human, and financial), cultural factors, and inefficient administrative policies (e.g., prolonged waiting times or disorganized patient flow). The consequences of wasta include inequities in healthcare, decreased healthcare professionals’ motivation and morale, and lower service quality.

### The social dynamics underpinning the utilization of wasta

The first major theme was the social powers employed to gain advantages through wasta. This theme encompassed the following subthemes: social status, wealth, social connections, and political affiliations.

Wasta based on social status or power was a pervasive reason for inequalities in accessing healthcare. For example, when asking about the sources of inequality in healthcare, a 30-year-old female COVID-19-patient from the West Bank narrated this incident which vividly illustrates how deeply ingrained wasta is in causing health inequalities in Palestine.

Interviewer: *Do you think there is equality in treatment?*

*No*,

*How?*

Participant: *“For example, during the first test, there was a person with a high social status who skipped the line. Wasta is deeply rooted in our society. They will not put it aside in times of crisis. On the contrary, this is a time for those who have wasta to use it”.*

*“During my third test, there were people who came from a government office and by chance I saw a friend of mine working there, and he told me that they entered from a backdoor*.

Interviewer: *They gave them special treatment?*

Participant: *Yes*.

Upon further probing, the patient revealed that the reasons behind those people receiving preferential treatment were social status, power, and wealth.

Interviewer*-This distinction for some people is it based on cultural background? Religious background? gender? What?*

Participant*- I feel it has more to do with one’s social status or power like are you rich, or have clout in the government.*

A female cancer patient from the West Bank confessed that her family members facilitated her transfer to a specialized hospital. Nepotism is the term applied to wasta when the mediators are family members rather than friends or acquaintances: *“Through nepotism, my family could transfer me to a specialized hospital in Jerusalem”.*

While patients unanimously conceded to the use of wasta in healthcare, health professionals did not always agree. A 30-year-old male surgeon who works at a government hospital disagreed that there are inequities in healthcare services: *“Sometimes some patients are given precedence over others but only based on the urgency of the case only. The services we provide for everybody equally, there is no discrimination in the government sector between citizens, except in cases where one case is more urgent than the other.”*

The aforementioned quotes offer insights into the robust correlation we observed between the region of residence and the utilization of wasta, as indicated by our quantitative models. It is conceivable that the cultural distinctions between these regions contribute to the heightened prevalence of wasta usage in Gaza and in camps when compared to the West Bank and urban areas.

### Wasta utilized via wealth and connections

This quote shared by a 52-year-old cancer patient in Gaza sheds light on the profound impact of financial resources on accessing healthcare services. It underscores how wealthier patients can secure special treatment through connections or financial means. The patient’s account unveils a complex ethical quandary existing within the healthcare field, particularly in developing nations.

Participant: *“Frankly, the financial situation was the most important. I received special treatment because I paid.”*

Interviewer: *Paid for whom?*

Participant: *“For everyone… my son, through his connections and his money, admitted me for special chest surgery. Thank God, he saved me but until now my consciousness is unsettled because I have no right to infringe on another person’s rights but our government is not providing and I do not blame the doctors”. I paid out of pocket to get my results quickly, but others cannot afford to pay for expedited results.”*

This quote captures an ethical dilemma confronting healthcare in developing countries. The high demands coupled with limited resources impede governments and healthcare providers from comprehensively meeting everyone’s requirements. Consequently, disparities rooted in financial wealth and social connections present intricate challenges that are difficult to adequately address.

### Wasta employed through political affiliations

The substantial impact of using wasta through political connections on healthcare experiences is exemplified by the following quotations:

A 37-year-old male patient from Gaza emphasized the importance of social connections and political affiliation in accessing superior healthcare quality and preferential treatment.

Interviewer: *Do you think people receive healthcare equally?*

Participant: *Not at all, it is all about relations (wasta)*.

Interviewer: *Do you mean patients without political affiliation will not receive health services?*

Participant: *He/she will take health services but incompletely.*

Interviewer: *How?*

Participant: *For instance, if someone is affiliated with a certain political faction and knows the hospital director, while another person from the general population is admitted, the one with political connections will receive comprehensive care, including a clean, air-conditioned room. The person from the general population will receive services, but of lesser quality.“*

In contrast to the above participant, a 32-year-old male patient from Gaza who was referred to the West Bank for treatment shared his experience:

*“In hospitals I did not see preferential treatments but when it comes to referrals if someone has a wasta like a father who is a political leader” then they will get expedited referrals.*

A 64-year-old male patient from Gaza agreed that belonging to a political faction confers advantages, significantly enhancing one’s ability to access and receive healthcare services.

*“As far as I know, yes the political affiliation has a large effect, this means this man belongs to a certain political faction he can take advantage, so knowing people has an effect.”*

These quotes emphasize the importance of addressing these inequities to ensure equal and fair healthcare opportunities for all individuals, regardless of their political affiliations or personal connections. It is crucial to address health inequities for several reasons. First, it is a matter of human rights, and violating this fundamental right perpetuates disparities based on factors such as race, ethnicity, socioeconomic status, and gender. Second, addressing health inequities is vital for the overall well-being and prosperity of societies. Finally, it is a moral imperative not to abandon vulnerable populations when they are in need.

### Predictors of using wasta in the healthcare settings

The study revealed the following factors that can drive patients to resort to wasta use: lack of financial resources, inefficient organizational processes, and tribal or collective cultures.

Lack of financial and human resources is one of the factors that can force patients to resort to wasta. A 71-year-old male diabetes patient from Jerusalem who was treated in Israel shared his perspective:

*“Personally, I never have seen wasta being used in Israeli institutions. Over there, in the West Bank yes there is wasta”.*

This patient explained that those with political affiliations in the West Bank receive treatment as first class. His perception of not encountering favoritism in Israel highlights the importance of healthcare resources and organizational efficiencies in obviating the need to resort to wasta. Israel spends 10 times more on resources compared to Palestine. When resources are abundant in terms of facilities, finances, and health professionals, patients’ access to care is facilitated, and the need to resort to wasta to access services is diminished.

The impact of scarcity of resources on service quality and equity was confirmed by several healthcare professionals and patients. A 30-year old surgeon at a government hospital said: *“We struggle with severe shortages in the medical staff, and the number of physicians and nurses is not enough to meet the needs of all patients.”*

The same sentiments were echoed by a 33-year-old male nurse who works at a government hospital who explained:

*“When you are responsible for 15 patients in a single shift and you strive to attend to all of their needs but you cannot, this becomes overwhelming. You barely have the time to prepare and administer the medications. We need to employ more staff. No time is left to educate the patients or provide emotional support.”*

The following quote from a 52-year old female patient agrees with the health professionals: *“Imagine you are one nurse serving 15 or more patients, a tragically enormous number; I do not blame the medical personnel, may God help them.”*

These quotations from the qualitative interviews complement the findings from quantitative analyses, which revealed a strong association between wasta usage and employment at government institutions. The qualitative insights delve further into the reasons behind the higher prevalence of wasta in government settings, specifically the limited resources that hinder equitable patient care.

The importance of human resources was also confirmed by a physician at a government hospital who noted that *“I worked at several government hospitals and I can attest that government hospitals with sufficient staffing tend to offer superior services, especially during periods of lower patient overload.”* This physician suggests that insufficient human resources at government hospitals are a more probable cause of challenges than inefficient administrative management at higher levels. The physician compared work experiences at different government hospitals operating under the same administrative protocols, highlighting the impact of staffing availability on service quality. However, this does not dismiss the possibility of administrative processes in the government sector requiring improvement. Other interviewees mentioned that the system of career advancement based on connections within government healthcare institutions demotivates employees significantly.

It is important to note that all obstacles to the smooth flow of operations provide a fertile ground for wasta to thrive. In addition to poverty and resource scarcity, inefficient administrative processes, prolonged waiting time, bureaucracies, and lack of cultural prohibitions force people to solve these problems by resorting to wasta and social networks.

### Consequences of using wasta in healthcare

The utilization of wasta in healthcare leads to outcomes such as disparities, reduced staff motivation, and compromised service quality. Through wasta and social connections some patients may gain unfair advantages over others, resulting in social inequities. Our interviews revealed that when staff are promoted and rewarded through wasta or social connections rather than for hard work, dedication, or performance, inequities become manifest. Such unfair practices cast a negative shadow on the institution and its services by eroding the morale and motivation of its personnel to excel in their careers. One physician explained: *“the other important point is the absence of incentives, a brilliant physician who is highly skilled in diagnosing and treating hard cases is never rewarded, encouraged, or acknowledged by superiors or the administration. Rewards and promotions are bestowed based on social connections rather than hard work, dedication and excellence. This culture of inequality demoralizes healthcare professionals and fosters mediocrity.”* This expressive statement illustrates how a promotion system based on wasta can detrimentally affect employees’ performance and consequently undermine the quality of services offered by the institution.

**Fig. 3:**
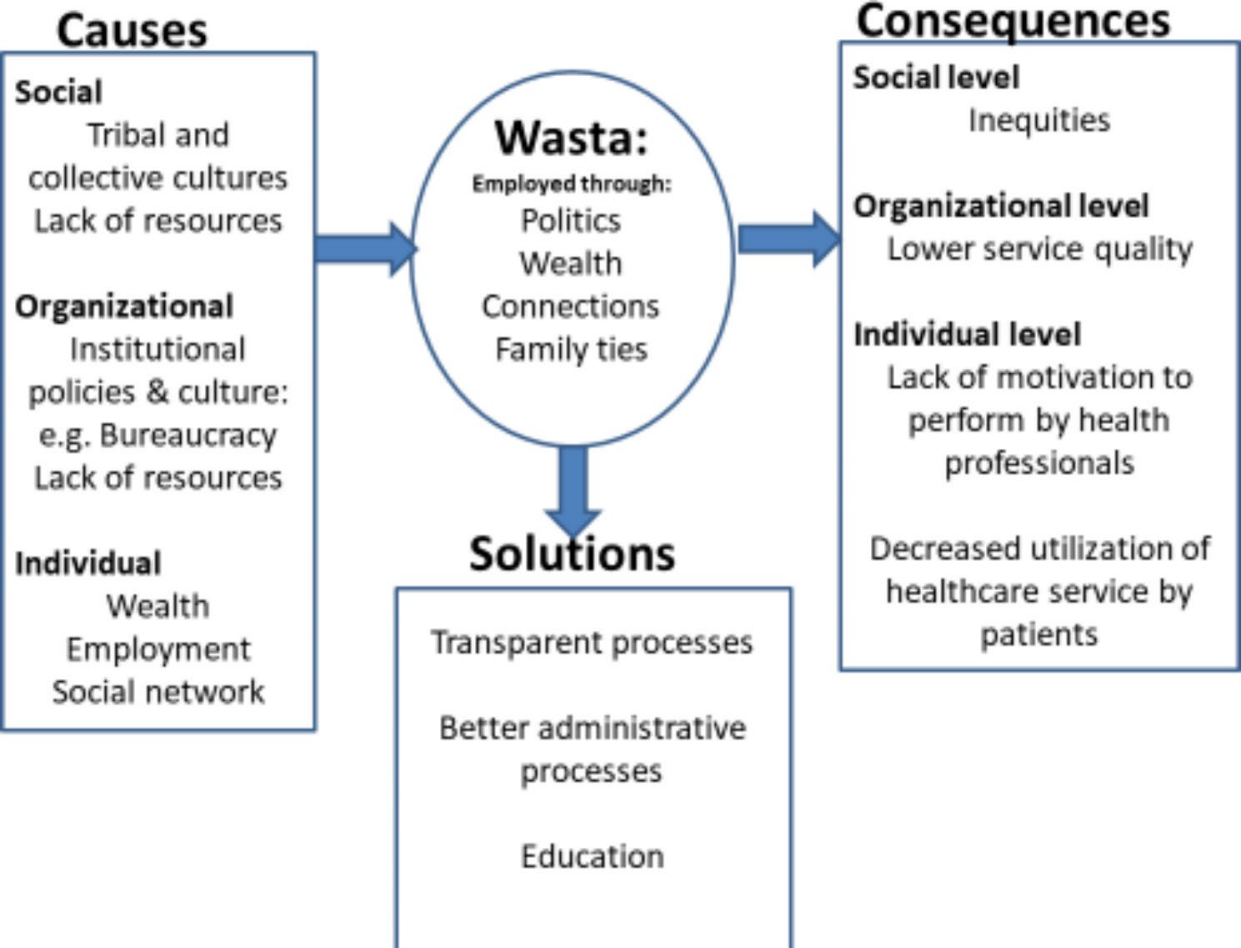
A conceptual framework of the predictors and consequences of wasta use and proposed solutions

This study significantly enriched the conceptual definition of wasta use in healthcare settings by elucidating the multifaceted mechanisms through which it manifests, its predictors, and its consequences. Figure 3 shows a summative conceptual framework of the main and subthemes that emerged in this study and in previous research. The central circle represents the factors through which wasta is employed. Wasta can be employed by utilizing social connections, political affiliations, family ties, and economic means. This study confirmed previous research about the role of organizational, social, and economic factors in causing wasta.

The box to the left of the circle shows the factors that precede, cause, or provide conditions for wasta to thrive. These include social norms such as a collective culture, tribal values of duty to serve and support kinship, and lack of resources that compel people to turn to wasta to fulfill needs unmet by official channels. The preceding factors also include organizational policies such as bureaucracies, inefficient processes (e.g. disorganized scheduling leading to long waiting times), and insufficient staffing and resources to meet patients’ needs. Finally, individual-level factors that can increase the chance of using wasta include being employed, being wealthy, and having a strong social network. Other researchers showed that people with extensive social networks are more likely to employ their friends and connections to gain social advantages through wasta [6, 12].

The box to the right of the circle shows the consequences of using wasta. Wasta can have negative consequences at the individual, organizational, and social levels. At the social level, wasta perpetuates inequities. At the organizational level, wasta can compromise service quality. At the individual level, health professionals who see that workplace promotion is based on wasta and connections will become demoralized and less motivated to excel in their work. Others found that where wasta is used to access healthcare, patients are less likely to seek and utilize healthcare services [2, 11].

The lower box in Fig. 3 presents proposed solutions to decrease the use of wasta in healthcare. Those include educating healthcare professionals on the ethical principles that underlie their profession, namely equity and beneficence through regular training, seminars, and workshops. Awareness can also be raised through the distribution of educational materials such as brochures, posters, and guidelines. Solutions also include changing organizational administrative policies and processes such as scheduling, staffing, and resource utilization.

## Discussion

Approximately one-third (34%) of respondents in our sample used wasta to get a health service in the last year, 39.3% from Gaza and 31.6% from the West Bank. This is lower than a 2021 public opinion poll in which 45% of Palestinians reported using wasta to obtain a service at public institutions [28]. The lower percentage in our study may be because our question was specific for health services. It is likely that the use of wasta varies by the type of service needed. Indeed, data from the Arab Barometer Surveys show that wasta is most commonly used to obtain employment. Approximately 88% of respondents from Palestine in these surveys reported obtaining employment through wasta [29]. In another poll conducted by Transparency International in 2019 about the use of wasta *for any service* in the MENA region, the highest use of wasta was in Lebanon (54%), followed by Palestine (39%), and Jordan (25%) [30].

### Gender

This study did not find a significant difference in the use of wasta to access health care by sex although previous research yielded mixed results. While some studies did not find differences in the use of wasta by sex, others found that men are more likely to use wasta than men. Alsarhan et al. (2021) did not find differences in wasta use by sex in Jordan [10]. Bailey (2012) in the UAE, Abdalla (2015) in the Arab Gulf states, and Tlaiss & Kauser (2010) in Lebanon found that women are at a disadvantage in terms of social capital, and networking opportunities compared to men [31–33]. Nuanced explorations, however, reveal that women use wasta through their family members rather than through their professional networks [6]. Women are also less likely to interact with men, limiting their opportunities to leverage wasta as effectively as men.

### Age, education, financial status, and employment

Analysis of data from Wave I of the Arab Barometer on use of wasta in five Arab countries (Yemen, Jordan, Palestine, Lebanon, and Algeria) showed a positive statistically significant relationship between use of wasta and employment status and income [7]. Employed and rich people are more likely to use wasta than poor and unemployed people.

Our findings indicate that education, financial status, and employment were significantly related to the use of wasta in the bivariate analysis. In the multivariate analysis, however, only employment remained statistically significant, indicating that the relationship between educational and financial status and wasta is a function of employment. These results corroborate previous research in Jordan revealing that the type of employment but not the amount of monthly salary is associated with wasta use [34]. The clear and strong relationship between employment and wasta use that has been confirmed in this and other studies requires further attention. It is likely that employment greatly enhances and expands a person’s social network and professional connections which they can then employ to gain social advantages.

### Region

We found that the place of residence (Gaza vs. West Bank or refugee camps vs. cities) is strongly associated with using wasta. The use of wasta was more common in Gaza and in refugee camps. These findings are consistent with previous research in Jordan that found highly significant correlations between the use of wasta and region of residence [30]. In Jordan, wasta use was more common in refugee camps and among Bedouin communities, which are more close-knit than cities. In Palestine, the higher prevalence of wasta in refugee camps and in Gaza is probably because these communities have faced disadvantages for generations, enduring various difficulties and injustices. It is possible that their perception, based on personal experiences or hearsay, is that utilizing wasta can help improve their situation.

Social relationships are different in refugee camps compared to cities and villages. In the camps, residents are bound by a common history of forced displacements and are living in crowded conditions. Political activism is stronger in camps. The situation is similar in Gaza where the population density is very high and political resistance is more intense than in the West Bank. In a similar study in Jordan, Jackson et al. (2019) mention that urbanization and the erosion of social ties in cities may weaken social ties and wasta and give rise to bribery in its place [4].

### The public (government) sector

The higher prevalence of wasta use among government employees that we found is consistent with previous reports of a higher prevalence of wasta use in the public sector in Jordan [35]. The current study underscores the importance of rewarding performance at work. The negative impact of focusing on relationships rather than performance negatively impacts employees’ motivations and morale. The quotes below from the Alsarhan and Valax (2020, p.9) qualitative study confirm the themes derived in our study [2].

*“If the way to be promoted is wasta, then you have no motivation to excel or to work on your credentials.”*

*“…Frustrated employees will transfer their frustration to the recipients of the services they are in contact with, thereby influencing the quality of services….”*

*“…The holders of high qualifications are at home, unemployed, while the less qualified individuals are in charge of responsibilities, the achievements and performance of public organizations is at the lowest level possible….”*

In our qualitative interviews, health professionals confirmed the above quotes, affirming the negative impact of wasta on healthcare institutions by saying that lack of incentives and rewards for highly performing physicians and nurses is a demotivating factor for them to excel and advance in their fields. Currently, career promotions in the government sector in Palestine are based on the duration of service not on achievements, performance, or work quality.

Our qualitative interviews also alluded to possible predictors of wasta use. One of these predictors is related to a lack of sufficient resources to meet the needs of the population. Resource scarcity will compel patients to resort to whatever means possible to access the much-needed services. Wasta, therefore, will have a better chance to thrive in low-resource settings than in settings where resources are abundant and available. This could explain why some participants mentioned that they have not experienced wasta in Israel but felt that it exists in the West Bank. Enormous disparities exist between the healthcare systems in Israel and Palestine in term of personnel, equipment, expenditure, and resources. For example, Israel’s healthcare expenditure per capita is 10 times higher than Palestine’s ($3,145 vs. $306 respectively) [36]. Around 3 hospital beds per 1000 population are available in Israel compared to only 1.3 beds per 1000 population in Palestine [36]. There are 5.7 nurses per 1000 people in Israel in contrast to only 1.9 nurses in Palestine and 4.6 physicians per1000 people in Israel as opposed to 2.7 in Palestine [36].

In summary, inequities lead to a situation where qualified personnel either remain unemployed or, if employed, are managed by less qualified personnel. Consequently, the overall quality of the organization’s services is compromised. Accountability, transparency, and the rule of law reduce the need for wasta [2].

### Limitations

When interpreting the results of this study, its limitations should be considered. First, the cross-sectional design precludes temporal and causal inferences. Secondg, the perceived discrimination questions are self-reported and subject to recall bias and social desirability bias as well as to personal interpretation of events. It is possible that some patients received better or worse services than others without them being aware of it. Future research may probe inequities in healthcare from the perspectives of healthcare providers. Despite these limitations, this study has several strengths. These include its large sample size, the inclusion of a diverse population, and the enrichment of the quantitative results with qualitative interviews.

## Recommendations

Given the negative consequences of wasta use on healthcare, we recommend addressing this challenging problem through training, raising awareness, transparency in operations, and institutional policies.

Training and raising awareness: The Palestinian Ministry of Health which is the largest provider of health services should invest in training medical professionals and senior managers on the principles of medical ethics, focusing especially on the principles of equality and equity in healthcare distribution and services. While equality means treating everybody equally, equity entails providing greater support for the poor, marginalized, and vulnerable. Healthcare providers should be educated not to offer preferential treatments or services to people based on their higher social status, employment status, wealth, or place of residence. In addition to training and educating health professionals, it is also important to raise awareness among the public about the negative impact of wasta. This can be done through media, social media, and community educational programs.

Transparency: Organizational policies should include a clear and open system to ensure transparency of work processes such as promotion policies, rewards, accountability mechanisms, and the oversight of ethical practices within both professional spheres and the organization. A system should be instituted where patients are able to voice their concerns and complaints through complaint boxes, exit surveys, and patient feedback. The exceptional service providers can be identified through audits or patient satisfaction surveys. Incentives could take the shape of rewards, prizes, or acknowledgments, granted to healthcare providers and/or institutions demonstrating exceptional performance in delivering their services.

In addition to patient surveys, it is also important to conduct regular health professionals’ surveys to understand their evaluation of the system they work within and the services they provide. Such surveys will help in identifying problems rapidly. Regular surveys and monitoring of the healthcare system should assess the critical aspects of quality, affordability, equity, and ethical delivery, including use of wasta. Additionally, intervention and longitudinal research to assess changes over time and the effectiveness of interventions is essential for evidence-based decisions and policies.

Administrative policies: several policies can help in reducing the need to use wasta in healthcare. These include reducing the bureaucratic procedures to access healthcare, reducing waiting times, and organizing scheduling and patient flow to reduce the need to resort to wasta to overcome these barriers. Healthcare organizations should also improve their resource management processes, increase their staffing, and invest in technologies to increase productivity.

## Conclusion

An approach that focuses on optimizing processes for efficiency, alleviating financial strains, implementing uncompromising accountability measures, and instituting comprehensive educational and training programs for health professionals and the public are potent solutions to curbing the reliance on wasta within the healthcare domain. This holistic strategy should dissolve institutional inefficiencies and tackle deeply ingrained cultural norms that perpetuate unethical practices. As societies embrace this multifaceted framework, they forge a path toward healthcare systems that stand as beacons of equity, accessibility, and unwavering ethical principles.

### Electronic supplementary material

Below is the link to the electronic supplementary material.

Supplementary Material 1

## Data Availability

The datasets used and analyzed during the current study are available from the corresponding author upon reasonable request.
