# Supplementary material for: A mixed methods study of using wasta in healthcare services in Palestine: predictors, consequences and proposed solutions
Source: BMC Health Serv Res. 2023 Oct 19;23:1123. doi: 10.1186/s12913-023-10114-5 (PMC10585732; doi:10.1186/s12913-023-10114-5)
Supplement: Supplementary file 1 — Supplementary Material 1 [file 12913_2023_10114_MOESM1_ESM.docx]

**Qualitative interviews with patients**

1. Describe your perceptions of healthcare in your region?
2. Describe your experience with your visit to the health center, physician for your condition
3. What is your understanding of your health rights?
4. In your opinion, how equitable and valuable are healthcare services in your area? Can you tell us stories; give examples that happened with you?
5. Have you heard of someone gaining advantageous access to health services through Wasta?
   1. If yes, what kind of advantage?

Prompts and probes if interviewees did not mention:

- - 1. Referral to Israeli hospitals
    2. Referral to non-governmental hospitals
    3. Preferential treatment at local hospitals
    4. Skipping lines (less waiting time)
    5. Admission to hospital
    6. Better treatment by nurses
    7. Political affiliation
    8. Other: ??

1. How do you cope with difficulties?

**Qualitative interviews with health professionals**

1. What are the most critical challenges/obstacles/problems you face in your practice?
2. What solutions you recommend?
3. What are your views about the fairness of the Palestinian healthcare model in terms of access, availability, and affordability? Especially for marginalized groups
4. What is your opinion of the quality of the healthcare services in your region?
5. Describe your thoughts on efficiency: maximizing benefits and minimizing waste?
6. What would improve quality, equity, and access?
7. How does political conflict affect your work? And the service for patients?
8. How do you cope with these challenges?
